# A clinical guideline for the Iranian women and newborns in the postpartum period

**DOI:** 10.1186/s12913-024-11026-8

**Published:** 2024-05-01

**Authors:** Leila Abdoli Najmi, Sakineh Mohammad-Alizadeh-Charandabi, Fatemeh Abbasalizadeh, Haniyeh Salehi Poormehr, Fariba Pashazade, Mojgan Mirghafourvand

**Affiliations:** 1 Department of Midwifery, Faculty of Nursing and Midwifery, Tabriz University of Medical Sciences, Tabriz, Iran; 2 Department of Midwifery, Faculty of Nursing and Midwifery, Tabriz University of Medical Sciences, Tabriz, Iran; 3 Women’s Reproductive Health Research Center, Tabriz University of Medical Sciences, Tabriz, Iran; 4 Research Center for Evidence-Based Medicine, Iranian EBM Center: A Joanna Briggs Institute Center of Excellence, Tabriz University of Medical Sciences, Tabriz, Iran; 5 Research Center for Evidence-Based Medicine, Iranian EBM Center, Tabriz University of Medical Sciences, Tabriz, Iran; 6 Social Determinants of Health Research Center, Tabriz University of Medical Sciences, Tabriz, Iran

**Keywords:** Clinical guideline, Cultural adaptation, Postpartum

## Abstract

**Background:**

The postpartum is a vital period for women, newborns, spouses, parents, caregivers, and families. Regarding the importance of postpartum care and the lack of comprehensive and up-to-date clinical guidelines in the country of Iran, the postpartum clinical guidelines have been adapted.

**Methods:**

Cultural adaptation was conducted in three stages. In the first stage, the adaptation team was formed and the process was approved. During the second stage, a systematic literature review was conducted using international databases to identify English-language clinical guidelines published within the last 10 years. Out of 17 guidelines and documents initially selected, 5 guidelines meeting the inclusion and exclusion criteria and published within the last 5 years were chosen following a thorough review by the search team. In the secondary selection, the guidelines were investigated by two subject-matter experts based on AGREE II Checklist, and regarding the high evaluation score obtained by the WHO Recommendations on Postnatal Care of the Mother and Newborn (2022), and the National Institute for Health and Care Excellence (NICE,2021) guideline for postnatal care were selected for cultural adaptation. In the third stage, the opinions of experts from all over the country were collected and scored using the Delphi method, and a final guideline was formulated.

**Results:**

The adapted postpartum clinical guideline has offered 56 recommendations. The recommendations are categorized into four major themes including mother care, newborn care, health system and health promotion interventions and post caesarean care.

**Conclusion:**

Applying evidence-based recommendations for the care of mothers and babies in the postpartum period will enhance the health system, promote the provision of care after vaginal and caesarean births, and ensure a positive postnatal experience for mothers, fathers, babies, and families.

**Supplementary Information:**

The online version contains supplementary material available at 10.1186/s12913-024-11026-8.

## Background

The postpartum period is critical for the long-term physical and mental health of mothers and children [1]. Quality care during the early days and weeks after childbirth significantly influences their experiences [2]. During this phase, women adapt to physical, mental, and social changes, which can present significant challenges such as insomnia, fatigue, breastfeeding issues, stress, mental health concerns, reduced sexual desire, and urinary incontinence. [3]. Socio-economic and cultural factors can influence mothers’ experiences, emotions, and behaviors during early motherhood [4]. Additionally, the support and encouragement provided to mothers by healthcare staff during childbirth, in hospital settings, at home, and through peer support can significantly influence their health and well-being [5].

Despite its importance, evidence suggests that postpartum care has been undervalued and under-resourced [6]. Studies indicate that many women are dissatisfied with the postpartum care they receive. For instance, a survey in England revealed that 50% of mothers felt they lacked adequate help, support, and information on newborn feeding [4]. Furthermore, in the United States, 40% of women do not attend their postpartum checkups, leading to challenges in managing chronic conditions, accessing effective contraception, and increasing the risk of short inter-pregnancy intervals and preterm delivery, particularly among disadvantaged communities [7].

In Iran, postpartum care often receives less attention compared to the antenatal period, both in terms of quality and quantity [8]. Research indicates that only 30% of mothers in developing countries receive adequate postpartum care, with approximately 70% expressing dissatisfaction with the care in Iran [9–11].

The American College of Obstetricians and Gynecologists advocates for continuous, personalized postpartum care to optimize the health of mothers and newborns, emphasizing that postpartum checkups should not be limited to a single visit at 6–8 weeks after delivery [7]. Similarly, the WHO recommends postpartum care within 24 h after childbirth, followed by at least three additional visits, aiming to improve maternal and newborn health [12].

Postpartum care plays a vital role in continuous care for mothers, newborns, and children, contributing to the achievement of sustainable development goals in reproductive health, such as reducing maternal and infant mortality [13]. However, the lack of attention to maternal health needs during the postpartum period contributes to one-third of maternal deaths, highlighting the need for improved clinical knowledge and technology for long-term prevention, particularly in underserved areas [14, 15].

Evidence-based guidelines for postpartum care can mitigate mid- and long-term complications, inform clinical management, and contribute to policy-making and unified care across healthcare departments and professions [12, 16]. The adaptation of clinical guidelines is essential to ensure their relevance and feasibility in local contexts, requiring significant resources, expertise, and coordination involving multidisciplinary experts, continuous supervision, and evaluation [17, 18].

Authentic and comprehensive clinical guidelines, such as the WHO Recommendations on Postnatal Care of the Mother and Newborn and the NICE guideline for postnatal care, provide a basis for routine care for mothers and newborns, with the potential for standardizing postnatal services through adaptation and utilization of international clinical guidelines [16, 19].

Considering the absence of comprehensive and current postnatal guidelines in Iran, as well as the WHO’s suggestion for the adaptation and utilization of clinical guidelines, this study aims to provide evidence-based recommendations for enhancing postnatal care in Iran, taking into account the sociocultural and healthcare system context of the country [12, 20].

## Methods

This study is the first phase of a multi-phase research that its protocol has already been published [21]. It is a multi-stage developmental research using systematic review and qualitative methods. It is aimed at the cultural adaptation of postnatal clinical guidelines in Tabriz University of Medical Sciences in 2023. The ADAPTE method has been used for the cultural adaptation of the clinical guidelines in the present study. Generally, ADAPTE includes three stages [18, 22]:

### First phase: set-up

During the set-up phase, the adaptation team was formed. The cultural adaptation team consists of 6 people (two reproductive health specialists, a perinatologist, a PhD in evidence-based studies, a PhD student in midwifery, and a senior expert in Medical Library and Information Science). The topic selection criteria were set. Also, the feasibility of adaptation of the clinical guideline was evaluated based on the availability of authentic international postnatal clinical guidelines, and then, the adaptation plan was prepared.

### Second phase: adaptation (search, and evaluation of the clinical guidelines)

Since the team aimed to adapt a clinical guideline for postnatal care, the search to find suitable clinical guidelines was conducted. A PIPOH-based search strategy was used to find the clinical guidelines and other relevant documents. The keyword “postpartum” was searched for in the Mesh and the terms “Postpartum Period” and “Postnatal Care” which were related to the searched keyword were retrieved. No Mesh equivalents were found for the keyword “Guideline”, so the search was conducted using the ‘Publication Type’ and ‘title/abstract’. The search for the relative keywords was conducted on the related websites (Table 1) in a 10-year period. In this phase, after removing duplicates, 17 guidelines and documents were selected. The selected guidelines are available as a supplementary file. Based on inclusion criteria (using the English language, availability of the full manuscript of the guideline, routine postpartum care, and being the latest or most common manuscript), and exclusion criteria (the target group not being the same and the topic background not matching), after the initial evaluations of the retrieved guidelines by the search team, 5 guidelines that were published within the last 5 years (Table 2) were primarily selected, which were evaluated and scrutinized for the second time. As the secondary selection, the guidelines were evaluated by two thematic experts, using the AGREE II Checklist. The AGREE II includes 23 appraisal criteria (items) organized within six domains. These domains are scope and purpose, stakeholder involvement, rigor of development, clarity of presentation, applicability, and editorial independence. Each criterion is given a score ranging from 1 (totally disagree) to 7 (totally agree). The score of each domain is calculated by the sum of the scores given to the criteria of that domain and standardizing the total score based on the maximum obtainable score for that domain. The quality score for all six domains was calculated [23, 24].

**Table 1:** The reliable sites to search for clinical guidelines

Title	URL
PubMed by limiting the search to clinical guidelines	www.pubmed.gov
National Guideline clearinghouse	http://www.guideline.gov
TRIP database	www.tripdatabase.com
The National Institute for Health and Care Excellence (NICE)	http://www.nice.org.uk
New Zealand Guidelines Group	http://www.nzgg.org.nz
Ontario Guidelines Advisory Committee (GAC) Recommended Clinical Practice Guidelines	http://www.gacguidelines.ca
National Guidelines Clearinghouse (NGC)	http://www.guideline.gov
MD Consult	http://www.mdconsult.com
G-I-N)) Guidelines International Network	http://www.g-i-n.net
Agency for Health Care Policy and Research	http://www.ahrq.gov/clinic/cpgonline.htm
Canadian Medical Association InfoBase	http://mdm.ca/cpgsnew/cpgs/index.asp
Directory of evidence- based information Websites	http://132.203.128.28/edicine/repertoire/repertoire.asp
*h*Scottish Intercollegiate Guidelines Network SIGN	http://www.sign.ac.uk
Australian National Health and Medical Research Council clinical practice guidelines (NHMRC)	http://www.nhmrc.gov.au
Australian Clinical Practice Guidelines	https://www.clinicalguidelines.gov.au
Infobase (CPG): Clinical Practice Guidelines – Canadian Medical Association infobase of clinical practice guidelines	https://www.cma.ca/En/Pages/clinical-practice-guidelines.aspx
The Cochran library	http://www3.interscience.wiley.com/cgibin/mrwhome/106568753/HOME
WHO | World Health Organization	https://www.who.int

**Table 2:** Characteristics of the included guidelines

**Country**	Guideline group	Short Name	Title	Year
**CANADA**	Public Health Agency of Canada	CMA	Postpartum care	2020
**USA**	American College of Obstetricians and Gynecologist	ACOG	Optimizing postpartum care. ACOG Committee Opinion No 736	2018
**France**	French College of Gynecologists and Obstetricians	CNGOF	Postpartum practice: guidelines for clinical practice from the French College of Gynaecologists and Obstetricians	2016
**International**	World Health Organization	WHO	WHO recommendations on maternal and newborn care for a positive postnatal experience	2022
**UK**	National Institute for Health and Care Excellence	NICE_194_	Postnatal care	2021

Regarding the high evaluation scores obtained by the WHO Recommendations on Postnatal Care of the Mother and Newborn (2022), and the National Institute for Health and Care Excellence (NICE) guideline for postnatal care (2021) (Table 3), these guidelines were selected for cultural adaptation. The recommendations for the preparation of the draft of the clinical guideline were selected and formulated in the form of a table.

**Table 3:** AGREE II domain scores for the included guidelines

**Organization**	**Scope and purpose**	**Stakeholder involvement**	**Rigor of development**	**Clarity of presentation**	Applicability	**Editorial independence**	Overall guideline assessment
	Percent	Percent	Percent	Percent	Percent	Percent	Percent
**WHO (2022)**	100	100	100	100	97.9	100	Strongly recommended
**NICE (2021)**	100	100	95	95	95	95	Strongly recommended
**CMA**	57	40	20	80	20	0	Recommended with alterations
**-2020**							
**ACOG (2018)**	47	60	30	52	18	60	Recommended with alterations
**CNGOF**	45	45	55	45	15	57	Recommended with alterations
**-2016**							

### Third phase: finalization (reviewing the target users and formulation of the final manuscript)

To reach a consensus on the clinical advantage and feasibility of cultural adaptation, the Delphi technique [25] was used to survey experts from different regions of the country via email. Eighteen experts from a variety of specialties, including reproductive health, obstetrics, midwifery, health policy, Evidence Based Medicine (EBM) and pediatrics, were invited to participate (Table 4). The panel of experts scored the recommendations in terms of the feasibility of cultural adaptation (low, medium, and high). The views and opinions of these experts were scored and summarized using a 9-item Likert scale. These points were scored from 1 to 9. Scores above 7 meant the approval of the recommendation, scores between 5 and 7 needed modifications in a second phase of Delphi, and scores lower than 4 meant the rejection of the recommendation. The most common definition of consensus, which is the agreement percentage, has been used for clinical advantage. An above-75% agreement was considered as the consensus threshold [26].

**Table 4:** Profile of cultural adaptation experts

Team experts	
Mojgan Mirghafourvand	Professor of Reproductive Health, Tabriz University of Medical Sciences, Tabriz, Iran
Sakineh Mohammad‑Alizadeh‑Charandabi	Professor of Reproductive Health, Tabriz University of Medical Sciences, Tabriz, Iran
Fatemeh Abbasalizadeh	Professor of Obstetrics and Gynecology Tabriz University of Medical Sciences, Tabriz, Iran
Haniyeh Salehi Poormehr	Assistant Professor of Neuroscience Research Center for Evidence-based Medicine, Tabriz University of Medical Sciences, Tabriz, Iran
Leila Abdoli Najmi	PhD Student of midwifery, Tabriz University of Medical Sciences, Tabriz, Iran
Fariba Pashazade	Senior expert in Medical Library & Information Science Research Center for Evidence‑Based Medicine, Iranian EBM Center, Tabriz University of Medical Sciences, Tabriz, Iran
**Panel experts**	
Mahin Kamalifard	Assistant Professor of Midwifery, Tabriz University of Medical Sciences, Tabriz, Iran
Solmaz Ghanbari-Homaie	Assistant Professor of Midwifery Education, Tabriz University of Medical Sciences, Tabriz, Iran
Parvin Abedi	Professor of Midwifery Education Ahvaz Jundishapur University of Medical Sciences,Iran
Fatemeh Bakouei	Associate Professor of Reproductive Health Babol University of Medical Sciences
Azita Tiznobaik	Assistant Professor of Reproductive Health Hamadan University of Medical Sciences
Zahra Behboodi Moghadam	Professor of Reproductive Health Tehran University of Medical Sciences
Pouran Akhavan Akbari	Assistant Professor of Reproductive Health Ardabil University of Medical Science
Roghieh Bayrami	Assistant Professor of Reproductive Health Urmia University of Medical Sciences
Manijhe Mostafa Gharehbaghi	Professor of Neonatal-Perinatal Medicine Tabriz University of Medical Sciences, Tabriz, Iran
Neda Kabiri	Assistant Professor of Health Policy Research Center for Evidence-based Medicine
Mina Iravani	Associate Professor of Reproductive Health Ahvaz Jundishapur University of Medical Sciences
Elham Rezaei	Assistant Professor of Reproductive Health, Tabriz University of Medical Sciences, Tabriz, Iran
Nahid Jahani Shoorab	Assistant Professor of Reproductive Health Mashhad University of Medical Sciences
Farzaneh Soltani	Associate Professor of Reproductive Health Hamadan University of Medical Science,Iran
Fahimeh Ranjbar	Assistant Professor of Reproductive Health Iran University of Medical Sciences

The clinical guideline was sent to authorities (Ministry of Health and Medical Education) for consultation and their views were applied to it. Finally, the culturally adapted guideline was formulated after being summarized, judged, and agreed upon by the panel of experts. The evidence grades of the recommendations in this guideline were based on the sum of evidence strength according to the reference clinical guidelines and the opinions of the panel of experts (Table 5).

**Table 5:** Definition of level of evidence

The validity of the recommendation	A
High performance	B
Intermediate or optional	C
Lack of recommendation or lack of sufficient evidence (based on expert opinions)	D

## Results

After the Delphi process, 56 clinical recommendations (Table 6) were developed in the form of care, prevention, treatment, education, and promotion of the health system themes. These recommendations were produced by a panel of experts from different regions of Iran and took into account the cultural, socio-economic, and health system constraints of the country. Out of those recommendations, 43 recommendations aligned with the WHO’s guidelines while 13 recommendations aligned with the NICE guidelines. As some recommendations in these guidelines overlapped, the WHO’s guideline were used as the main guideline, and the NICE guideline was used for some specific recommendations related to post-caesarean procedures. The recommendations are designed to improve maternal and child health outcomes and address the specific needs and contexts of these groups in Iran.

**Table 6:** Recommendations of adapted clinical guidelines for postpartum period

Care category	Recommendation	Level of evidence
**A. MATERNAL CARE**		
**Maternal assessment**	1. “All postpartum women should have regular assessment of vaginal bleeding, uterine tonus, fundal height, temperature and heart rate (pulse) routinely during the first 24 h, starting from the first hour after birth. In the first hour after delivery”, blood pressure should be measured every 15 min to one hour, then every half hour to two hours according to the country’s protocol. Urine void should be documented within 6 h At each subsequent postnatal contact beyond 24 h after birth, enquiries should continue to be “made about general well-being and assessments made regarding the following: micturition and urinary incontinence, bowel function, healing of any perineal wound, headache, fatigue, back pain, perineal pain and perineal hygiene, breast pain and uterine tenderness and lochia.”	A
**HIV catch-up testing**	2. For women who are HIV negative or have an unknown HIV status, who are considered to be at high risk of contracting HIV (such as people whose sexual partners are infected, or who themselves or their spouses are addicted to injecting drugs), If they have not done the test at the first pregnancy visit or the re-test at the end of pregnancy in the third trimester, it is necessary to do an HIV test	A
**Interventions for common physiological signs and symptoms**		
**Local cooling for perineal pain relief**	3. “Local cooling, such as with ice packs or cold pads, can be offered to women in the immediate postpartum period for the relief of acute pain from perineal trauma sustained during childbirth, based on a woman’s preferences and available options”	A
**Oral analgesia for perineal pain relief**	4. “Oral paracetamol (acetaminophen) 325 mg every 6 h is recommended as first-line choice when oral analgesia is required for the relief of postpartum perineal pain”	A
**Pharmacological relief of pain due to uterine cramping/involution**	5. “Oral non-steroidal anti-inflammatory drugs (NSAIDs) can be used when analgesia is required for the relief of postpartum pain due to uterine cramping after childbirth, based on a woman’s preferences, the clinician’s experience with analgesics and availability”	A
**Postnatal pelvic floor muscle training for pelvic floor strengthening**	6. “For postpartum women, starting routine pelvic floor muscle training (PFMT) after childbirth for the prevention of postpartum urinary and faucal incontinence is recommended”	B
**Non-pharmacological interventions to treat postpartum breast engorgement**	7. “For treatment of breast engorgement in the postpartum period, women should be counselled and supported to practice responsive breastfeeding, good positioning and attachment of the baby to the breast, expression of breastmilk, and the use of warm or cold compresses, based on a woman’s preferences”	A
**Preventive measures**		
**Non-pharmacological interventions to prevent postpartum mastitis**	8. “For the prevention of mastitis in the postpartum period, women should be counselled and supported to practice responsive breastfeeding, good positioning and attachment of the baby to the breast, hand expression of breastmilk, and the use of warm or cold compresses, based on a woman’s preferences”	A
**Pharmacological interventions to prevent postpartum mastitis**	9. “Routine oral or topical antibiotic prophylaxis for the prevention of mastitis in the postpartum period is not recommended”	A
Care category	Recommendation	Level of evidence
**Prevention of postpartum constipation**	10 a. “Dietary advice and information on factors associated with constipation should be offered to women for the prevention of postpartum constipation”	A
	10 b. “Routine use of laxatives for the prevention of postpartum constipation is not recommended”	A
**Prevention of maternal peripartum infection after uncomplicated vaginal birth**	11. “Routine antibiotic prophylaxis for women with uncomplicated vaginal birth is not recommended”	A
**Mental health interventions**		
**Screening for postpartum depression and anxiety**	12. “Screening for postpartum depression and anxiety using a validated instrument is recommended and should be accompanied by diagnostic and management services for women who screen positive”	A
**Prevention of postpartum depression and anxiety**	13. “Psychosocial and/or psychological interventions during the antenatal and postnatal period are recommended to prevent postpartum depression and anxiety”	A
**Nutritional interventions and physical activity**		
**Postpartum oral iron and folate supplementation**	14. “Oral iron supplementation, either alone or in combination with folic acid supplementation, may be provided to postpartum women for 6–12 weeks following childbirth for reducing the risk of anemia in settings where gestational anemia is of public health concern”	A
**Postpartum vitamin A supplementation**	15. “Vitamin A supplementation in postpartum women for the prevention of maternal and infant morbidity and mortality is not recommended”	A
**Physical activity and sedentary behaviour**	16 a. “All postpartum women without contraindication should: • Undertake regular physical activity throughout the postpartum period; • Do at least 150 min of physical activity throughout the week for substantial health benefits; and • Incorporate a variety of physical and muscle-strengthening activities; adding gentle stretching may also be beneficial”	A
	16 b. “Postpartum women should limit the amount of time spent being sedentary. Replacing sedentary time with physical activity of any intensity (including light intensity) provides health benefits”	A
**Contraception**		
**Postpartum contraception**	17. Providing of information and services related to the interval between pregnancies is recommended	B
**B. NEWBORN CARE**		
**Assessment of the newborn for danger signs**	18. “The following signs should be assessed during each postnatal care contact, and the newborn should be referred for further evaluation if any of the signs is present: not feeding well; history of convulsions; fast breathing (breathing rate > 60 per minute); severe chest in-drawing; no spontaneous movement; fever (temperature > 37.5 °C); low body temperature (temperature < 35.5 °C); any jaundice in first 24 h after birth, or yellow palms and soles at any age” “The parents and family should be encouraged to seek health care early if they identify any of the above danger signs between postnatal care visits”	A
**Universal screening for abnormalities of the eye**	19. “Universal newborn screening for abnormalities of the eye is recommended and should be accompanied by diagnostic and management services for children identified with an abnormality”	A
**Universal screening for hearing impairment**	20. “Universal newborn hearing screening (UNHS) with otoacoustic emissions (OAE) or automated auditory brainstem response (AABR) is recommended for early identification of permanent bilateral hearing loss (PBHL) UNHS should be accompanied by diagnostic and management services for children identified with hearing loss”	A
**Universal screening for neonatal hyperbilirubinaemia**	21 a. “Universal screening for neonatal hyperbilirubinaemia by transcutaneous bilirubinometer (TcB) is recommended at health facility discharge”	A
	21 b. “There is insufficient evidence to recommend for or against universal screening by total serum bilirubin (TSB) at health facility discharge”	A
**Preventive measures**		
**Timing of first bath to prevent hypothermia and its sequelae**	22. “The first bath of a term, healthy newborn should be delayed for at least 24 h after birth. Mothers should be taught how to bathe their babies before discharge from the hospital”	A
**Use of emollients for the prevention of skin conditions**	23. “Routine application of topical emollients in term, healthy newborns for the prevention of skin conditions is not recommended”	A
**Application of chlorhexidine to the umbilical cord stump for the prevention of neonatal infection**	24 a. “Clean, dry umbilical cord care is recommended”	A
	24 b. “Daily application of 4% chlorhexidine (7.1% chlorhexidine digluconate aqueous solution or gel, delivering 4% chlorhexidine) to the umbilical cord stump in the first week after birth is recommended only in settings where harmful traditional substances (e.g. animal dung) are commonly used on the umbilical cord. In case of using chlorhexidine, it will be necessary to comply with the conditions to prevent irritation of the healthy skin of the baby”	A
**Sleeping position for the prevention of sudden infant death syndrome**	25. “Putting the baby to sleep in the supine position during the first year is recommended to prevent sudden infant death syndrome (SIDS) and sudden unexpected death in infancy (SUDI)”	A
**Immunization for the prevention of infections**	26. “Newborn immunization should be promoted as per the latest existing WHO recommendations for routine immunization”	A
**Nutrition interventions**		
**Neonatal vitamin A supplementation**	27 a. “Routine neonatal vitamin A supplementation is not recommended to reduce neonatal and infant mortality”	A
	27 b. “In settings with recent (within the last five years) and reliable data that indicate a high infant mortality rate (greater than 50 per 1000 live births) and a high prevalence of maternal vitamin A deficiency (≥ 10% of pregnant women with serum retinol concentrations < 0.70 μmol/L), providing newborns with a single oral dose of 50 000 IU of vitamin A within the first three days after birth may be considered to reduce infant mortality”	A
**Vitamin D supplementation for breastfed, term infants**	28. “Vitamin D supplementation in breastfed, term infants is recommended for improving infant health outcomes in our context”. In our country, mothers should be advised to start vitamin A + D drops for infants from the 3rd to the 5th day after birth	A
**Infant growth and development**		
**Whole-body massage**	29. “Gentle whole-body massage may be considered for term, healthy newborns for its possible benefits to growth and development”	A
**Early childhood development**	30 a. “All infants and children should receive responsive care between 0 and 3 years of age; parents and other caregivers should be supported to provide responsive care”	A
	30 b. “All infants and children should have early learning activities with their parents and other caregivers between 0 and 3 years of age; parents and other caregivers should be supported to engage in early learning with their infants and children”	A
	30 c. “Support for responsive care and early learning should be included as part of interventions for optimal nutrition of newborns, infants and young children”	A
	30 d.” Psychosocial interventions to support maternal mental health should be integrated into early childhood health and development services”	A
**Exclusive breastfeeding**	31. “All babies should be exclusively breastfed from birth until 6 months of age. Mothers should be counselled and provided with support for exclusive breastfeeding at each postnatal contact’	A
**Protecting, promoting and supporting breastfeeding in facilities providing maternity and newborn services**	32 a. “Facilities providing maternity and newborn services should have a clearly written breastfeeding policy that is routinely communicated to staff and parents”	A
	32 b. “Health-facility staff who provide infant feeding services, including breastfeeding support, should have sufficient knowledge, competence and skills to support women to breastfeed”	A
**C. Health systems and health promotion interventions**		
**Schedules for postnatal care contacts**	33. “A minimum of four postnatal care contacts is recommended. If birth is in a health facility, healthy women and newborns should receive postnatal care in the facility for at least 24 h after birth. If birth is at home, the first postnatal contact should be as early as possible within 24 h of birth. At least three additional postnatal contacts are recommended for healthy women and newborns, between48 and 72 h, between 7 and 14 days, and during week six after birth”	A
**Length of stay in health facilities after birth**	34. “Care for healthy women and newborns in the health facility is recommended for at least 24 h after vaginal birth”	A
**Criteria to be assessed prior to discharge from the health facility after birth**	35. “Prior to discharging women and newborns after birth from the health facility to the home, health workers should assess the following criteria to improve maternal and newborn outcomes: • The woman’s and baby’s physical well-being and the woman’s emotional well-being; • The skills and confidence of the woman to care for herself and the skills and confidence of the parents and caregivers to care for the newborn; and • The home environment and other factors that may influence the ability to provide care for the woman and the newborn in the home, and care-seeking behavior”	B
**Approaches to strengthen preparation for discharge from the health facility to home after birth**	36. “Information provision, educational interventions and counselling are recommended to prepare women, parents and caregivers for discharge from the health facility after birth to improve maternal and newborn health outcomes, and to facilitate the transition to the home. Educational materials, such as written/digital education booklets, pictorials for semi-literate populations and job aids should be available”	A
**Home visits for postnatal care contacts**	37. “Home visits during the first week after birth by skilled health personnel or a trained community health worker are recommended for the postnatal care of healthy women and newborns. Where home visits are not feasible or not preferred, outpatient postnatal care contacts are recommended”	B
**Midwifery continuity of care**	38. “Midwife-led continuity-of-care (MLCC) models, in which a known midwife or small group of known midwives supports a woman throughout the antenatal, intrapartum and postnatal continuum, are recommended for women in settings with well-functioning midwifery programs”	B
**Task sharing components of postnatal care delivery**	39 a. “Task sharing the promotion of health-related behaviors for maternal and newborn health to a broad range of cadres, including lay health workers, auxiliary nurses, nurses, midwives and doctors, is recommended”	B
	39 b. “Task sharing the provision of recommended postpartum contraception methods to a broad range of cadres, including auxiliary nurses, nurses, midwives and doctors, is recommended”	B
**Recruitment and retention of staff in rural and remote areas**	40. “Policy-makers should consider a bundle of interventions covering education, regulation, incentives and personal and professional support to improve health workforce development, attraction, recruitment and retention in rural and remote areas”	B
**Involvement of men in postnatal care and maternal and newborn health**	41. “Interventions to promote the involvement of men during pregnancy, childbirth and after birth are recommended to facilitate and support improved self-care of women, home care practices for women and newborns, and use of skilled care for women and newborns during pregnancy, childbirth and the postnatal period, and to increase the timely use of facility care for obstetric and newborn complications These interventions are recommended, provided they are implemented in a way that respects, promotes and facilitates women’s choices and their autonomy in decision-making, and that supports women in taking care of themselves and their newborns”	B
**Home-based records**	42. “The use of home-based records, as a complement to facility-based records, is recommended for the care of pregnant and postpartum women, newborns and children, to improve care-seeking behaviour, men’s involvement and support in the household, maternal and child home care practices, infant and child feeding, and communication between health workers and women, parents and caregivers”	C
**Digital targeted client communication**	43. “WHO recommends digital targeted client communication for behaviour change regarding sexual, reproductive, maternal, newborn and child health, under the condition that concerns about sensitive content and data privacy are adequately addressed”	B
**NICE Recommendations**		
**Principles of care**	44. “When caring for a woman who has recently given birth, listen to her and be responsive to her needs and preferences”	A
**Bed sharing**	45 a. “Discuss with parents’ safer practices for bed sharing, including: • Making sure the baby sleeps on a firm, flat mattress, lying face up (rather than face down or on their side) • Not sleeping on a sofa or chair with the baby • Not having pillows or duvets near the baby • Not having other children or pets in the bed when sharing a bed with a baby”	A
	45 b. “Strongly advise parents not to share a bed with their baby if their baby was low birth weight or if either parent: • Has had 2 or more units of alcohol • Smokes • Has taken medicine that causes drowsiness • Has used recreational drugs”	A
**Promoting emotional attachment**	46 a. “Before and after the birth, discuss the importance of bonding and emotional attachment with parents, and the approaches that can help them to bond with their baby”	A
	46 b. “Encourage parents to value the time they spend with their baby as a way of promoting emotional attachment, including: • Face-to-face interaction • Skin-to-skin contact • Responding appropriately to the baby’s cues”	A
	46 c. “Discuss with parents the potentially challenging aspects of the postnatal period that may affect bonding and emotional attachment, including: • the woman’s physical and emotional recovery from birth • Experience of a traumatic birth or birth complications • Fatigue and sleep deprivation • Feeding concerns • Demands of parenthood”	B
	46 d. “Recognise that additional support in bonding and emotional attachment may be needed by some parents who, for example: • Have been through the care system • Have experienced adverse childhood events • Have experienced a traumatic birth • Have complex psychosocial needs”	B
**Care after caesarean birth**		
**Pain management after caesarean birth**	47 a. “Offer oral morphine sulfate to women who have received spinal or epidural anesthesia for caesarean birth. If the woman cannot take oral medication (for example, because of nausea or vomiting), offer intravenous, intramuscular or subcutaneous morphine”	A
	47 b. Consider intravenous patient-controlled analgesia (PCA) using morphine for women who have had a general anesthetic for caesarean birth. If intravenous PCA is not acceptable to the woman, or the pain is less severe, consider oral morphine sulfate or Diclofenac suppositories or intramuscular Pethidine or tramadol or Ketorolac Consider laxatives for women taking opioids, for the prevention of constipation	A
	47 c. “Use paracetamol and, unless contraindicated, a non-steroidal anti-inflammatory drug (for example, ibuprofen) in combination after caesarean birth, to reduce the need for opioids, and to allow them to be stepped down and stopped as early as possible” • Pain relief after caesarean birth can be done according to the hospital protocol	A
	47 d. “For women with severe pain after caesarean birth, when other pain relief is not sufficient: perform a full assessment to exclude other causes for the pain (for example, sepsis, hemorrhage, urinary retention)”	A
**Early eating and drinking after caesarean birth**	48. “Mothers will be NPO for 8 to 24 h after surgery, depending on the case, If women are recovering well after caesarean birth and do not have complications, they can eat and drink as normal”	A
**Urinary catheter removal after caesarean birth**	49. “Offer removal of the urinary bladder catheter once a woman is mobile after a regional anesthetic for caesarean birth, but no sooner than 12 h after the last 'top-up’ dose”	A
**Respiratory physiotherapy after caesarean birth**	50. “Do not offer routine respiratory physiotherapy (Encouragement to take deep breaths or cough) to women after a caesarean birth under general anesthesia as it does not improve respiratory outcomes (for example, coughing, phlegm, body temperature, chest palpation or auscultatory changes)”	C
**Discharge**	51. “Offer women who are recovering well, are apyrexial and do not have complications after caesarean birth, discharge from hospital after 24 h and follow up at home, as this is not associated with more readmissions for babies or mothers”	A
**Wound care**	52 a. “Consider negative pressure wound therapy after caesarean birth for women with a BMI of 35 kg/m2 or more to reduce the risk of wound infections When using standard (not negative pressure) wound dressings after caesarean birth take into account that: No type of wound dressing has been shown to be better than another at reducing the risk of wound infections The dressings are removed 24 to 48 days after the operation”	B
	52b. “Ensure caesarean birth wound care includes: Removing standard dressings 6 to 24 h after the caesarean birth Specific monitoring for fever, assessing the wound for signs of infection (such as increasing pain, redness or discharge), separation or dehiscence Encouraging the woman to wear loose, comfortable clothes and cotton underwear Bathe daily (gently cleaning and drying the wound daily)”	A
**Resuming activities and discharge home**	53. “Inform women who have had a caesarean birth that they can resume activities such as driving a vehicle, carrying heavy items, formal exercise and sexual intercourse when they feel they have fully recovered from the caesarean birth (including any physical restrictions or pain)”	A
**Management of symptoms**	54 a. “When caring for women who have had a caesarean birth who have urinary symptoms, consider possible diagnoses of: urinary tract infection stress incontinence (occurs in about 4% of women after caesarean birth) urinary tract injury (occurs in about 1 per 1,000 caesarean births) urinary retention”	A
	54 b. “When caring for women who have had a caesarean birth who have heavy and/or irregular vaginal bleeding, consider whether this is more likely to be because of endometritis than retained products of conception, and manage accordingly”	A
	54 c. “When caring for women who have had a caesarean birth, discuss that after a caesarean birth they are not at increased risk of depression, post-traumatic stress symptoms, pain on sexual intercourse, fecal incontinence or difficulties with breastfeeding”	A
	54 d. “Pay particular attention to women who have respiratory symptoms (such as cough or shortness of breath) or leg symptoms (such as painful swollen calf), as women who have had a caesarean or vaginal birth may be at increased risk of thromboembolic disease (both deep vein thrombosis and pulmonary embolism)”	A
**Follow-up**	55. “Inform the woman’s GP if follow-up investigations are needed after discharge from hospital (for example, a repeat full blood count if there has been a large amount of blood loss), and include details of the plan or course of action if the results are abnormal”	B
**Pregnancy and childbirth after caesarean birth**	56 a. “When advising about the mode of birth after a previous caesarean birth, consider: Maternal preferences and priorities The risks and benefits of repeat planned caesarean birth The risks and benefits of planned vaginal birth after caesarean birth, including the risk of unplanned caesarean birth”	B
	56 b. “Inform women who have had up to and including repeat caesarean births that the risk of fever, bladder injuries and surgical injuries does not vary with planned mode of birth, but that the risk of uterine rupture is higher for planned vaginal birth”	B
	56 c. “Offer women planning a vaginal birth who have had a previous caesarean birth: Electronic fetal monitoring during labour Care during labour in a unit where there is immediate access to caesarean birth and on-site blood transfusion services”	B
	56 d. “Pregnant women with both previous caesarean birth and a previous vaginal birth should be informed that they have an increased likelihood of having a vaginal birth than women who have had a previous caesarean birth but no previous vaginal birth”	A

The recommendations are categorized into four major themes: (1) mother care (recommendations 1–17), (2) newborn care (recommendations 18–32), and (3) health system and health promotion interventions (recommendations 33–43). (4) post caesarean care (recommendations 46–56).

The mother care section, including different themes regarding maternal assessment, preventive interventions, mental health interventions, nutritional and physical activity interventions, and information and services for interval between pregnancies. Additionally, there were 10 recommendations specifically addressing the needs of women who had caesarean delivery, and 1 recommendation about the delivery experience and 2 recommendations about child safety and emotional connection with the child.

The neonatal section covers various areas such as physiological examination, preventive interventions, nutritional interventions, newborn growth and development, and breastfeeding.

The health system and health promotion section provides 11 recommendations addressing important areas such as health promotion, policy formulation, monitoring and evaluation, and evidence-based decision-making.

## Discussion

The postpartum is a very sensitive period for the mothers and the newborn. The lack of comprehensive and up-to-date clinical guidelines in Iran has created a significant gap in the provision of postnatal care. Cultural adaptation of clinical guidelines for postpartum is a vital step to ensure that mothers, newborns, spouses, parents, and families receive suitable and evidence-based care in this critical period.

Among the evaluated guidelines, the WHO Recommendations on Postnatal Care of the Mother and Newborn (2022), and the National Institute for Health and Care Excellence (NICE, 2021) guideline for postnatal care obtained the highest scores in six appraisal domains with AGREE II. Also, in the study by Yang et al. [27], these guidelines obtained the highest score in terms of the use of the latest evidence, methodological quality, and the use of a multi-disciplinary team for the formulation of the recommemdations. The AGREE II is used to formulate or adapt the guideline. It was also used as a credible reference to evaluate the quality of clinical guidelines [28]. This tool has been used in various studies, as a standard benchmark for investigation of the quality of clinical guidelines [27, 29, 30].

The WHO Recommendations on Postnatal Care of the Mother and Newborn are about the common postnatal care for healthy mothers and newborns. This clinical guideline is a comprehensive collection of 55 recommendations that have provided the components of postnatal care in three main categories: a) maternal care (24 recommendations), b) newborn care (19 recommendations), and c) healthcare system and health promotion interventions (12 recommendations). Since the WHO’s guideline only covers vaginal birth and provides no recommendations for cesarean birth, and considering the high statistics of cesarean in our country [31, 32], the recommendation by the panel of experts who emphasized that the adapted guideline should be comprehensive and also cover the cesarean delivery, the National Institute for Health and Care Excellence (NICE) guideline for postnatal care was also used for post-cesarean care recommendations.

During the initial evaluation of the WHO’s guidelines by the cultural adaptation team, 5 maternal care recommendations were removed (No 3, 9, 15, 16, 17). Due to the low prevalence of tuberculosis, schistosomiasis, parasites (worms) and HIV in our country, recommendations related to tuberculosis screening, prevention of schistosomiasis, drug prevention of parasites (Preventive anthelminthic treatment), and Oral pre-exposure prophylaxis for HIV prevention were removed. Medical prevention of mastitis with subcutaneous oxytocin (No. 9) and vitamin A consumption in pregnancy (No. 21), which were not recommended by the WHO, were removed according to experts’ opinion.

The WHO’s recommendations on immediate postnatal evaluation of mother (No. 1) and HIV screening (No. 2) were formulated by some modifications after the first stage of Delphi and with the consensus of the experts. According to the WHO’s guidelines (No. 7), it is not recommended to do exercise to strengthen the pelvic floor muscles, but with the consensus of the experts (over 75% agreement), this recommendation was added to the adapted guideline.

Regarding the population policies of the country, and based on the opinions of the experts, the WHO’s recommendation on the provision of contraception information and services (No. 24) was changed into ‘provide information and services related to the interval between pregnancies. According to experts’ opinion, mothers should be advised to start vitamin A + D drops for infants from the 3rd to the 5th day after birth (No. 28).

In terms of post-cesarean pain relief in the NICE guideline, the ‘protocols of the related hospital for pain relief’ and names of some common pain relievers were added based on the experts’ recommendations (No. 47 b). In the recommendation of the NICE clinical guide regarding vaginal childbirth after cesarean section, according to experts, labor induction was omitted, because it is not performed in our country in mothers with a history of cesarean section.

The WHO recommendations related to health system and health promotion interventions, specifically recommendations 35, 36, 37, 38, 39, 41, 42 and 43, would require specific infrastructure to be effectively implemented in Iran. These recommendations include the criteria to be assessed prior to discharge after birth, midwifery continuity of care, home-based records, digital targeted client communication, and cultural considerations related to men’s involvement in some regions of the country. Although these recommendations may pose challenges to implement in the current state of the health system, experts agree that they should not be removed from the guideline, but rather should serve as a long-term goal to move the health system towards creating the necessary infrastructure to implement these recommendations effectively in the future.

## Conclusion

By implementing evidence-based recommendations for the care of mothers and babies in the postpartum period, the healthcare system will be strengthened in order to promote the provision of care after vaginal and caesarean births. Additionally, a positive postnatal experience will be ensured for mothers, fathers, babies, and families. By adopting these recommendations, the overall health outcomes of mothers and babies during this critical period can be improved. The experts emphasize the importance of integrating these recommendations into healthcare policies and practices in order to promote a comprehensive and evidence-based approach to maternal and child health in Iran.

### Supplementary Information

Additional file 1

## Data Availability

Not applicable.

## Abbreviations

WHO: World Health Organization
SDGs: Sustainable development goals
ADAPTE: Group name for adaption a Clinical guideline
AGREE: Appraisal of Guidelines for Research and Evaluation
NICE: National Institute for Health and Care Excellence

## References

[1] Belemsaga DY, Kouanda S, Goujon A, Kiendrébéogo J, Duysburgh E, Degomme O (2015). A review of factors associated with the utilization of healthcare services and strategies for improving postpartum care in Africa. Afrika Focus.

[2] Bick D, MacArthur C, Winter H. Postnatal Care E-Book. 2nd ed. London: Churchill Livingstone; 2008.

[3] Aber C, Weiss M, Fawcett J (2013). Contemporary women’s adaptation to motherhood: the first 3 to 6 weeks postpartum. Nurs Sci Q.

[4] Redshaw M, Rowe R, Hockley C, Brocklehurst P. Recorded delivery: a national survey of women’s experience of maternity care 2006: National Perinatal Epidemiology Unit NPEU. Oxford: Joshua Horgan; 2007. Available from: https://www.npeu.ox.ac.uk/assets/downloads/maternity-surveys/reports/Maternity-Survey-Report-2006-Recorded-Delivery.pdf. Accessed 30 Apr 2024.

[5] Barnes J, Ram B, Leach P, Sylva K (2007). Factors associated with negative emotional expression: a study of mothers of young infants. J Reprod Infant Psychol.

[6] Bhavnani V, Newburn M. Left to your own devices: The postnatal care experiences of 1260 first-time mothers. National Childbirth Trust; 2010. https://www.nct.org.uk/sites/default/files/related_documents/PostnatalCareSurveyReport5.pdf.

[7] American College of Obstetricians and Gynecologists (2018). Optimizing postpartum care. ACOG Committee opinion no. 736. Obstet Gynecol.

[8] Sakkaky M, Khairkhah M, Hosseini AF (2010). The effect of home visit after cesarean delivery on exclusive breastfeeding in neonatal period. Iran J Nurs.

[9] Mojalli M, Basiri Moghadam M, Shamshiri M (2010). Effectiveness of instructional environment and related factors on breastfeeding function of mothers. Intern Med Today.

[10] Mansour Lamadah S, El-Nagger N (2014). Mothers’ satisfaction regarding quality of postpartum nursing care and discharge teaching plan at ain shams maternity and gynecological hospital. Int J Curr Res.

[11] Mohseni MA, Bahadoran P, Abedi H (2009). The quality of postpartum care from mothers’ viewpoint. Hakim Res J.

[12] World Health Organization (2022). WHO recommendations on maternal and newborn care for a positive postnatal experience.

[13] Every Woman Every Child. Global Strategy for Women’s, Children’s and Adolescents Health 2016–2030. 2015. Available online. https://www.who.int/life-course/partners/global-strategy/globalstrategyreport2016-2030-lowres.pdf. Accessed 11 Jan 2020.

[14] WHO, UNICEF, UNFPA, World Bank Group and the United Nations Population Division. Trends in maternal mortality 2000 to 2017; 2019. Available from: https://www.unfpa.org/resources/trends-maternal-mortality-2000-2017-executive-summary. Accessed 30 Apr 2024.

[15] World Health Organization (2016). Standards for improving quality of maternal and newborn care in health facilities.

[16] World Health Organization (2018). WHO Recommendations: Intrapartum Care for a Positive Childbirth Experience.

[17] Ministry of Health, Treatment and Medical Education. Department of Standardization and Compilation of Clinical Guidelines. Guidelines Adapting. Available from: https://hetas.behdasht.gov.ir/uploads/422/2021/Mar.pdf: Accessed 5 May 2022.

[18] Harrison MBLF, Légaré Légaré F, Graham ID, Fervers B (2010). Adapting clinical practice guidelines to local context and assessing barriers to their use. CMAJ.

[19] The National Institute for Health and Care Excellence. Postnatal care. Available from:. https://www.nice.org.uk/guidance/ng194/resources/postnatal-care-pdf-66142082148037 Accessed 10 Jul 2023.

[20] OliaeiManesh A, Shirvani A, SalehiZelani Gh, Rabanikhah F, MousaGholizadeh R, Nejati M (2013). National guidelines for clinical medicine (2).

[21] AbdoliNajmi L, Mohammad-Alizadeh-Charandabi S, Jahanfar S, Abbasalizadeh F, SalehiPoormehr H, Mirghafourvand M (2023). Adaptation and implementation of clinical guidelines on maternal and newborn postnatal care in Iran: study protocol. Reprod Health.

[22] Mwangi N, Gachago M, Gichangi M, Gichuhi S, Githeko K, Jalango A (2018). Adapting clinical practice guidelines for diabetic retinopathy in Kenya: process and outputs. Implement Sci.

[23] Brouwers MC, Kho ME, Browman GP, Burgers JS, Cluzeau F, Feder G, et al., Development of the AGREE II paovoia. https://doi.org/ttsaCE.

[24] Rashidian A, Yousefi-Nooraie R (2012). Development of a Farsi translation of the AGREE instrument, and the effects of group discussion on improving the reliability of the scores. J Eval Clin Pract.

[25] Diamond IR, Robert C, Feldman BM, Pencharz PB, Ling SC, Moore AM (2014). Defining consensus: a systematic review recommends methodologic criteria for reporting of Delphi studies. J Clin Epidemiol.

[26] Habibi A, Izadyar S, Sarafrazi A (2014). Delphi technique theoretical framework in qualitative research. IJES.

[27] Yang M, Yue W, Han X (2022). Postpartum care indications and methodological quality:a systematic review of guidelines. J Public Health.

[28] Moradi-Joo M, Olyaeemanesh A, Akbari-Sari A, Rayegani SM (2022). Adaptation Frameworks for Clinical Guidelines and Proposing a Framework for Iran: A Review and Comparative Study. Med. J. Islam.

[29] Tara FDR, Saghafi N, Mirteimouri M, Ghooshkhanei H,  Soltanifar A, Salehi M, Dadgar S (2013). Management of Post-Partum Hemorrhage (Clinical Guideline). Iran J Obstet Gynecol Infertil.

[30] Coronado-Zarco R, de León AOG, Faba-Beaumont MG (2021). Adaptation of clinical practice guidelines for osteoporosis in a Mexican context. Experience using methodologies ADAPTE, GRADE-ADOLOPMENT, and RAND/UCLA. J Clin Epidemiol.

[31] Jafarzadeh AHM, Hasanshahi G, Rezaeian M, Vazirinejad R, Aminzadeh F, Sarkoohi A (2019). Cesarean or Cesarean Epidemic?. Arch Iran Med.

[32] Rafiei M, Ghare MS, Akbari M, Kiani F, Sayehmiri F, Sayehmiri K (2018). Prevalence, causes, and complications of cesarean delivery in Iran: A systematic review and meta-analysis. Int J Reprod Biomed.

