# Supplementary material for: A clinical guideline for the Iranian women and newborns in the postpartum period
Source: BMC Health Serv Res. 2024 May 1;24:563. doi: 10.1186/s12913-024-11026-8 (PMC11064303; doi:10.1186/s12913-024-11026-8)
Supplement: Supplementary file 1 — Additional file 1 [file 12913_2024_11026_MOESM1_ESM.docx]

| **Supplementary file:** Selected guidelines from the systematic literature review | | | | |
| --- | --- | --- | --- | --- |
|  | **Country** | **Guideline group** | **Title** | **Year** |
| **1.** | **WHO** | World Health Organization | WHO recommendations on  postnatal care of the mother and newborn. | 2013 |
| **2.** | **UK** | National Institute for Health and Care  Excellence | Antenatal and postnatal mental health: clinical management and  service guidance. | 2014b |
| **3.** | **WHO** | World Health Organization | Postnatal Care for Mothers and Newborns | 2015 |
| **4.** | **WHO** | World Health Organization | Global strategy for women’s, children’s and  adolescents’ health (2016–2030). New York  (NY): Every Woman Every Child | 2015 |
| **5.** | **France** | French College of Gynecologists and Obstetricians | Post-partum: Guidelines for clinical practice | 2015 |
| **6.** | **France** | French College of Gynecologists and Obstetricians | Postnatal visit: Routine and particularity after complicated pregnancy--Guidelines for clinical practice | 2015 |
| **7.** | **France** | French College of Gynecologists and Obstetricians | Postpartum practice: guidelines for clinical practice from the French  College of Gynaecologists and Obstetricians | 2016 |
| **8.** | **WHO** | World Health Organization | Standards for improving quality of maternal and  newborn care in health facilities | 2016 |
| **9.** | **USA** | American College of Obstetricians and  Gynecologist | Committee Opinion No. 666: Optimizing Postpartum Care | 2016 |
| **10.** | **CANADA** | Public Health Agency of Canada | Family-centred maternity and newborn care in Canada | 2017 |
| **11.** | **USA** | American College of Obstetricians and Gynecologist | Optimizing postpartum care. ACOG Committee Opinion No 736 | 2018a |
| **12.** | **Australia** | COAG Health Council | Woman-centred care  Strategic directions for Australian maternity services | 2019 |
| **13.** | **USA** | American College of Obstetricians and  Gynecologist | No 742: postpartum pain management | 2018c |
| **14.** | **CANADA** | Public Health Agency of Canada | Postpartum care | 2020 |
| **15.** | **Australia** | Maternal, Neonatal & Gynaecology  Community of Practice  (state government) | Postnatal Care:  Routine care of the  well woman and  neonate | 2021 |
| **16.** | **UK** | National Institute for Health and Care  Excellence | Postnatal care | 2021 |
| **17.** | **WHO** | World Health Organization | WHO recommendations on maternal and newborn care for a positive postnatal experience | 2022 |
